# *N*-(4-Chloro­phen­yl)ethanimidamide

**DOI:** 10.1107/S1600536810011013

**Published:** 2010-03-27

**Authors:** Nubia Boechat, Warner B. Kover, Sabrina B. Ferreira, Solange M. S. V. Wardell, James L. Wardell, Edward R. T. Tiekink

**Affiliations:** a Fundaçao Oswaldo Cruz, Instituto de Tecnologia em Fármacos, Departamento de Síntese Orgânica, Manguinhos, CEP 21041250 Rio de Janeiro, RJ, Brazil; b Universidade Federal do Rio de Janeiro, Departamento de Química Orgânica, Instituto de Química, Cidade Universitária, 21949-900 Rio de Janeiro, RJ, Brazil; c CHEMSOL, 1 Harcourt Road, Aberdeen AB15 5NY, Scotland; d Centro de Desenvolvimento Tecnológico em Saúde (CDTS), Fundação Oswaldo Cruz (FIOCRUZ), Casa Amarela, Campus de Manguinhos, Av. Brasil 4365, 21040-900 Rio de Janeiro, RJ, Brazil; e Department of Chemistry, University of Malaya, 50603 Kuala Lumpur, Malaysia

## Abstract

A twisted conformation is found in the title compound, C_8_H_9_ClN_2_, with the ethanimidamide residue being twisted substantially to the benzene ring [dihedral angle = 66.54 (14)°]. The conformation about the C=N double bond [1.299 (3) Å] is *Z*. A two-dimensional array with a zigzag topology is formed in the crystal structure *via* N—H⋯N and N—H⋯Cl hydrogen-bonding inter­actions.

## Related literature

For background to the synthesis of *N*-(*p*-chloro­phen­yl)acetamidine and related *N*-aryl­acetamidines used as reagents in the formation of anti-leishmanial compounds, see: Shearer *et al.* (1997 ▶); Rousselet *et al.* (1993 ▶); Patai (1975 ▶). For background to leismaniasis, see: Ouellette *et al.* (2004 ▶); Croft *et al.* (2006 ▶); Ferreira *et al.* (2007 ▶); World Health Organization (2010 ▶).
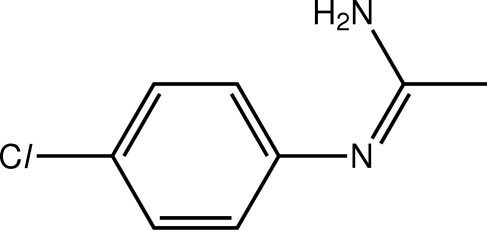


## Experimental

### 

#### Crystal data

C_8_H_9_ClN_2_
*M* *_r_* = 168.62
Orthorhombic, 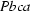

*a* = 9.6460 (9) Å
*b* = 9.0192 (4) Å
*c* = 19.3281 (5) Å
*V* = 1681.53 (18) Å^3^
*Z* = 8
Mo *K*α radiation
μ = 0.39 mm^−1^
*T* = 120 K
0.35 × 0.20 × 0.10 mm

#### Data collection

Nonius KappaCCD area-detector diffractometer
Absorption correction: multi-scan (*SADABS*; Sheldrick, 2007 ▶) *T*_min_ = 0.792, *T*_max_ = 1.000
14006 measured reflections
1924 independent reflections
1185 reflections with *I* > 2σ(*I*)
*R*_int_ = 0.081

#### Refinement

*R*[*F* ^2^ > 2σ(*F* ^2^)] = 0.047
*wR*(*F* ^2^) = 0.150
*S* = 1.05
1924 reflections
107 parameters
H atoms treated by a mixture of independent and constrained refinement
Δρ_max_ = 0.34 e Å^−3^
Δρ_min_ = −0.33 e Å^−3^

### 

Data collection: *COLLECT* (Hooft, 1998 ▶); cell refinement: *DENZO* (Otwinowski & Minor, 1997 ▶) and *COLLECT*; data reduction: *DENZO* and *COLLECT*; program(s) used to solve structure: *SHELXS97* (Sheldrick, 2008 ▶); program(s) used to refine structure: *SHELXL97* (Sheldrick, 2008 ▶); molecular graphics: *ORTEP-3* (Farrugia, 1997 ▶) and *DIAMOND* (Brandenburg, 2006 ▶); software used to prepare material for publication: *publCIF* (Westrip, 2010 ▶).

## Supplementary Material

Crystal structure: contains datablocks global, I. DOI: 10.1107/S1600536810011013/hg2664sup1.cif

Structure factors: contains datablocks I. DOI: 10.1107/S1600536810011013/hg2664Isup2.hkl

Additional supplementary materials:  crystallographic information; 3D view; checkCIF report

## Figures and Tables

**Table 1:** Hydrogen-bond geometry (Å, °)

*D*—H⋯*A*	*D*—H	H⋯*A*	*D*⋯*A*	*D*—H⋯*A*
N2—H1n⋯N1^i^	0.88 (3)	2.08 (3)	2.965 (3)	176 (3)
N2—H2n⋯Cl1^ii^	0.80 (3)	2.83 (3)	3.464 (2)	138 (3)

Symmetry codes: (i) 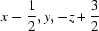; (ii) 
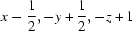
.

**Fig. 1.**
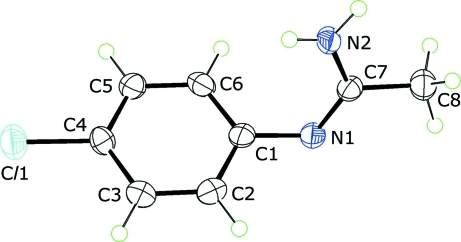
The molecular structure of (I) showing the atom-labelling scheme and displacement ellipsoids at the 50% probability level.

**Fig. 2.**
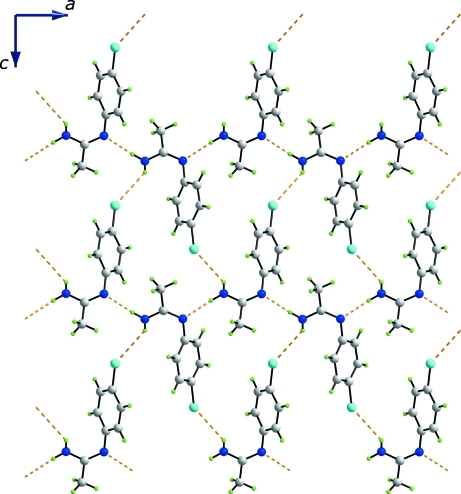
A view of a supramolecular array in (I) in the ac plane. The N–H···N and N–H···Cl hydrogen bonding interactions are shown as orange dashed lines. Colour code: Cl, cyan; N, blue; C, grey; and H, green.

## supplementary crystallographic information

### Comment

*N*-(*p*-Chlorophenyl)acetamidine and related *N*-arylacetamidines (Shearer *et al.* 1997; Rousselet *et al.* 1993; Patai, 1975) were synthesized for use as reagents in the formation of 5-(difluoromethyl)-2-methyl-1-(substituted-phenyl)-1*H*-imidazoles, which are active anti-leishmanial compounds (Ferreira *et al.*, 2007). Leishmaniasis is caused by several species of protozoan parasites transmitted by the bite of the female phlebotomine sand fly. This neglected disease is currently prevalent in four continents, being endemic in 88 countries, 72 of which are developing countries, threatening 350 millions worldwide (World Health Organization, 2010). The treatment of Leishmaniasis, currently, is dependent on old and highly toxic drugs (Croft *et al.*, 2006). In addition, the development of clinical resistance and the increase of co-infections leishmaniasis AIDS, in some countries is causing further worries. Thus, the development of new, efficient, and safe drugs for the treatment of this disease is imperative (Ouellette *et al.*, 2004; Croft *et al.*, 2006; Ferreira *et al.*, 2007). This contribution describes the synthesis and crystallographic characterisation of an *N*-(*p*-chlorophenyl)acetamidine derivative, (I).

The molecular structure of (I), Fig. 1, is twisted about the C1–N1 bond as seen in the value of the C2–C1–N1–C7 torsion angle of -118.6 (2) °; the dihedral angle formed between the benzene ring and ethanimidamide residue is 66.54 (14) °. The molecule has approximate mirror symmetry with the non-hydrogen atoms of the ethanimidamide lying on the putative plane and the benzene ring being bisected by the plane. The conformation about the C7═ N1 double bond [1.299 (3) Å] is *Z*.

The crystal packing is dominated by N–H···N and N–H···Cl hydrogen bonding interactions, Table 1. These lead to the formation of 22-membered {···HNH···ClC_4_NCNH···ClC_4_N···HNCN}_2_ synthons that are connected into supramolecular arrays in the *ac* plane, Fig. 2; these have a zig-zag topology.

### Experimental

To a stirred solution of *p*-chloroaniline (10.75 mmol) in acetonitrile (40 ml) was bubbled hydrogen chloride. A precipitate was formed immediately. The resulting suspension was refluxed and became homogeneous. Upon complete reaction, as shown by TLC, the mixture was rotary evaporated and the residue partitioned between CH_2_Cl_2_ and saturated aqueous NaHCO_3_. The aqueous layer was washed (3 times) with CH_2_Cl_2_, and the combined organic layers were dried over sodium sulfate, filtered, and the filtrate concentrated under reduced pressure to yield a white solid; yield 96%, m.p. 389–390 K. The sample used in the X-ray study was slowly grown from an ethanol solution of (I). IR (KBr, cm^-^1): 3451, 3295, 3079, 1640, 1586, 1482. ^1^H NMR (500 MHz, CDCl_3_): δ 1.99 (*s*, 3H, CH_3_); 4.53 (*br s*, 2H, 2); 6.77 (*d*, 2H, J = 8.0 Hz); 7.24 (*d*, 2H, J = 8.0 Hz) p.p.m. ^13^C NMR (125 MHz, CDCl_3_): δ 21.59 (CH_3_); 122.5 121.1; 128.6; 144.6; 155.3 (H_2_N—C=N) p.p.m. EI—MS (m/z): 168 (M^+^, 68%); 153 (M^+^-15, 38%); 127 (M^+^-41, 100%); 111 (M^+^ -57, 54%); 75 (M^+^-93, 42%).

### Refinement

The C-bound H atoms were geometrically placed (C–H = 0.95–0.98 Å) and refined as riding with *U_iso_*(H) = 1.2-1.5*U_eq_*(C). The positions of the N–H atoms were refined with *U_iso_*(H) = 1.2*U_eq_*(N).

### Crystal data

**Table:**

C_8_H_9_ClN_2_	*F*(000) = 704
*M**_r_* = 168.62	*D*_x_ = 1.332 Mg m^−^^3^
Orthorhombic, *P**b**c**a*	Mo *K*α radiation, λ = 0.71073 Å
Hall symbol: -P 2ac 2ab	Cell parameters from 2182 reflections
*a* = 9.6460 (9) Å	θ = 2.9–27.5°
*b* = 9.0192 (4) Å	µ = 0.39 mm^−^^1^
*c* = 19.3281 (5) Å	*T* = 120 K
*V* = 1681.53 (18) Å^3^	Block, colourless
*Z* = 8	0.35 × 0.20 × 0.10 mm

### Data collection

**Table:**

Nonius KappaCCD area-detector diffractometer	1924 independent reflections
Radiation source: Enraf Nonius FR591 rotating anode	1185 reflections with *I* > 2σ(*I*)
10 cm confocal mirrors	*R*_int_ = 0.081
Detector resolution: 9.091 pixels mm^-1^	θ_max_ = 27.5°, θ_min_ = 3.0°
φ and ω scans	*h* = −11→12
Absorption correction: multi-scan (*SADABS*; Sheldrick, 2007)	*k* = −11→9
*T*_min_ = 0.792, *T*_max_ = 1.000	*l* = −25→21
14006 measured reflections	

### Refinement

**Table:**

Refinement on *F*^2^	Primary atom site location: structure-invariant direct methods
Least-squares matrix: full	Secondary atom site location: difference Fourier map
*R*[*F*^2^ > 2σ(*F*^2^)] = 0.047	Hydrogen site location: inferred from neighbouring sites
*wR*(*F*^2^) = 0.150	H atoms treated by a mixture of independent and constrained refinement
*S* = 1.05	*w* = 1/[σ^2^(*F*_o_^2^) + (0.081*P*)^2^] where *P* = (*F*_o_^2^ + 2*F*_c_^2^)/3
1924 reflections	(Δ/σ)_max_ = 0.001
107 parameters	Δρ_max_ = 0.34 e Å^−^^3^
0 restraints	Δρ_min_ = −0.33 e Å^−^^3^

### Special details

**Table:**

Geometry. All s.u.'s (except the s.u. in the dihedral angle between two l.s. planes) are estimated using the full covariance matrix. The cell s.u.'s are taken into account individually in the estimation of s.u.'s in distances, angles and torsion angles; correlations between s.u.'s in cell parameters are only used when they are defined by crystal symmetry. An approximate (isotropic) treatment of cell s.u.'s is used for estimating s.u.'s involving l.s. planes.
Refinement. Refinement of *F*^2^ against ALL reflections. The weighted *R*-factor wR and goodness of fit *S* are based on *F*^2^, conventional *R*-factors *R* are based on *F*, with *F* set to zero for negative *F*^2^. The threshold expression of *F*^2^ > 2σ(*F*^2^) is used only for calculating *R*-factors(gt) etc. and is not relevant to the choice of reflections for refinement. *R*-factors based on *F*^2^ are statistically about twice as large as those based on *F*, and *R*- factors based on ALL data will be even larger.

### Fractional atomic coordinates and isotropic or equivalent isotropic displacement parameters (Å^2^)

**Table:**

	*x*	*y*	*z*	*U*_iso_*/*U*_eq_	
Cl1	0.43525 (8)	0.20450 (7)	0.43084 (3)	0.0430 (3)	
N1	0.36389 (19)	0.4659 (2)	0.71058 (10)	0.0308 (5)	
N2	0.1234 (2)	0.4244 (2)	0.70948 (11)	0.0305 (5)	
H1N	0.044 (3)	0.438 (3)	0.7313 (13)	0.037*	
H2N	0.124 (3)	0.379 (3)	0.6740 (14)	0.037*	
C1	0.3774 (2)	0.4032 (3)	0.64327 (12)	0.0274 (6)	
C2	0.4543 (2)	0.2746 (3)	0.63454 (14)	0.0316 (6)	
H2	0.4938	0.2272	0.6738	0.038*	
C3	0.4741 (3)	0.2144 (3)	0.56933 (13)	0.0317 (6)	
H3	0.5266	0.1260	0.5638	0.038*	
C4	0.4169 (2)	0.2837 (3)	0.51261 (13)	0.0286 (6)	
C5	0.3428 (3)	0.4144 (3)	0.51965 (12)	0.0342 (6)	
H5	0.3054	0.4627	0.4802	0.041*	
C6	0.3243 (3)	0.4736 (3)	0.58491 (13)	0.0348 (6)	
H6	0.2745	0.5638	0.5901	0.042*	
C7	0.2408 (2)	0.4770 (3)	0.73731 (13)	0.0269 (6)	
C8	0.2239 (3)	0.5538 (3)	0.80540 (14)	0.0367 (6)	
H8A	0.3153	0.5706	0.8261	0.055*	
H8B	0.1680	0.4919	0.8364	0.055*	
H8C	0.1774	0.6492	0.7983	0.055*	

### Atomic displacement parameters (Å^2^)

**Table:**

	*U*^11^	*U*^22^	*U*^33^	*U*^12^	*U*^13^	*U*^23^
Cl1	0.0537 (5)	0.0446 (5)	0.0307 (4)	0.0051 (3)	0.0060 (3)	−0.0034 (3)
N1	0.0210 (11)	0.0449 (13)	0.0264 (12)	−0.0010 (9)	−0.0007 (9)	−0.0032 (9)
N2	0.0205 (11)	0.0461 (14)	0.0248 (12)	−0.0033 (9)	0.0019 (9)	−0.0061 (10)
C1	0.0171 (11)	0.0372 (14)	0.0280 (14)	−0.0036 (10)	−0.0002 (10)	0.0003 (11)
C2	0.0286 (13)	0.0340 (14)	0.0321 (15)	−0.0010 (11)	−0.0040 (11)	0.0052 (11)
C3	0.0297 (13)	0.0288 (13)	0.0367 (16)	0.0031 (10)	0.0014 (11)	0.0001 (11)
C4	0.0284 (13)	0.0306 (15)	0.0266 (14)	−0.0016 (10)	0.0072 (10)	0.0012 (10)
C5	0.0327 (14)	0.0437 (16)	0.0263 (14)	0.0074 (11)	0.0018 (11)	0.0068 (11)
C6	0.0296 (14)	0.0403 (15)	0.0345 (15)	0.0119 (11)	0.0047 (12)	0.0019 (11)
C7	0.0233 (12)	0.0329 (13)	0.0246 (14)	−0.0026 (10)	−0.0006 (10)	0.0029 (10)
C8	0.0281 (13)	0.0512 (16)	0.0307 (14)	−0.0059 (12)	0.0014 (11)	−0.0066 (12)

### Geometric parameters (Å, °)

**Table:**

Cl1—C4	1.743 (3)	C3—C4	1.377 (4)
N1—C7	1.299 (3)	C3—H3	0.9500
N1—C1	1.425 (3)	C4—C5	1.385 (3)
N2—C7	1.340 (3)	C5—C6	1.381 (3)
N2—H1N	0.89 (3)	C5—H5	0.9500
N2—H2N	0.80 (3)	C6—H6	0.9500
C1—C2	1.387 (3)	C7—C8	1.496 (4)
C1—C6	1.393 (3)	C8—H8A	0.9800
C2—C3	1.386 (4)	C8—H8B	0.9800
C2—H2	0.9500	C8—H8C	0.9800
			
C7—N1—C1	118.52 (19)	C6—C5—C4	119.1 (2)
C7—N2—H1N	119.4 (17)	C6—C5—H5	120.5
C7—N2—H2N	121 (2)	C4—C5—H5	120.5
H1N—N2—H2N	119 (3)	C5—C6—C1	121.0 (2)
C2—C1—C6	118.7 (2)	C5—C6—H6	119.5
C2—C1—N1	119.5 (2)	C1—C6—H6	119.5
C6—C1—N1	121.6 (2)	N1—C7—N2	125.8 (2)
C3—C2—C1	120.8 (2)	N1—C7—C8	119.0 (2)
C3—C2—H2	119.6	N2—C7—C8	115.2 (2)
C1—C2—H2	119.6	C7—C8—H8A	109.5
C4—C3—C2	119.4 (2)	C7—C8—H8B	109.5
C4—C3—H3	120.3	H8A—C8—H8B	109.5
C2—C3—H3	120.3	C7—C8—H8C	109.5
C3—C4—C5	121.0 (2)	H8A—C8—H8C	109.5
C3—C4—Cl1	119.65 (19)	H8B—C8—H8C	109.5
C5—C4—Cl1	119.4 (2)		

### Hydrogen-bond geometry (Å, °)

**Table:**

*D*—H···*A*	*D*—H	H···*A*	*D*···*A*	*D*—H···*A*
N2—H1n···N1^i^	0.88 (3)	2.08 (3)	2.965 (3)	176 (3)
N2—H2n···Cl1^ii^	0.80 (3)	2.83 (3)	3.464 (2)	138 (3)

Symmetry codes: (i) *x*−1/2, *y*, −*z*+3/2; (ii) *x*−1/2, −*y*+1/2, −*z*+1.
